# The neurocognitive and neuropsychiatric manifestations of Susac syndrome: a brief review of the literature and future directions

**DOI:** 10.1007/s10072-024-07672-9

**Published:** 2024-07-02

**Authors:** Rebecca Koncz, Miranda J. Say, Andrew Gleason, Todd A. Hardy

**Affiliations:** 1 The University of Sydney Specialty of Psychiatry, Concord, NSW Australia; 2 Department of Psychiatry, Concord Repatriation General Hospital, Concord, NSW Australia; 3 Department of Psychology, Concord Repatriation General Hospital, Concord, NSW Australia; 4 Department of Consultation-Liaison Psychiatry, Concord Repatriation General Hospital, Concord, NSW Australia; 5 Florey Institute of Neuroscience and Mental Health, The University of Melbourne, Melbourne, VIC Australia; 6 Department of Neuroscience, Central Clinical School, Monash University, Melbourne, VIC Australia; 7 Department of Neurology, Concord Repatriation General Hospital, Concord, NSW Australia; 8 Brain and Mind Centre, University of Sydney, Camperdown, NSW Australia

**Keywords:** Susac syndrome, Encephalopathy, Neurocognitive, Neuropsychiatric

## Abstract

Encephalopathy is part of the clinical triad of Susac syndrome, but a detailed understanding of the neurocognitive and neuropsychiatric profile of this condition is lacking. Existing literature indicates that cognitive deficits range in severity from subtle to profound. Executive function and short-term recall are affected frequently. Psychiatric manifestations may be absent or may include anxiety, mood disorders or psychosis. If psychiatric phenomena develop during the disease course, it can be hard to disentangle whether symptoms directly relate to the pathology of Susac syndrome or are secondary to treatment-related side effects. In this article, we review what is known about the cognitive and psychiatric morbidity of Susac syndrome and identify areas where knowledge is deficient. Importantly, we also provide a framework for future research, arguing that better phenotyping, understanding of pathophysiology, evaluation of treatments on cognitive and psychiatric outcomes, and longitudinal data capture are vital to improving patient outcomes.

## Background

Susac syndrome is a condition that affects the brain, eye and ear and is characterised by a clinical triad of encephalopathy, branch retinal artery occlusions and sensorineural hearing loss [1]. It is a rare disease with an annual incidence of 0.024 to 0.13/100,000 [2, 3], but it is an important differential diagnosis for several more common conditions including multiple sclerosis and stroke [4, 5]. Only 13–30% of patients present with the full clinical triad which can make diagnosis difficult [6].

A seminal review of all published cases of Susac syndrome in 2013 determined that 76% (230 out of 304 cases) presented with encephalopathy [7]. More specifically, 48% of total cases presented with cognitive impairment, 16% with emotional disturbance, 15% with behaviour change, 12% with apathy or personality change, and 10% with psychosis [7]. Neuropsychiatric symptoms are considered important enough to the diagnosis that new cognitive and/or behavioural changes are deemed to be core indicators of “brain involvement”, along with new focal neurological symptoms and/or headache, according to the European Susac Consortium (EuSaC) diagnostic criteria for Susac syndrome [8].

Despite the high rates of neuropsychiatric features reported, the cognitive and psychiatric manifestations are not well characterised. Patients can develop new neuropsychiatric symptoms over the course of their disease, and it is not always clear if these symptoms arise due to underlying disease activity, treatment with corticosteroids, or psychiatric conditions, for example secondary to the stress of living with a chronic and relapsing disease. There are also uncertainties about how best to treat neuropsychiatric symptoms in Susac syndrome, to what extent these symptoms are reversible, and what features of the disease might inform the cognitive and psychiatric prognosis.

In this paper we review what is known of the cognitive and psychiatric symptoms that occur in Susac syndrome, identify knowledge gaps, and propose directions for future research.

### Cognitive impairment in Susac syndrome

During the last decade, several case series describing the phenomenology of Susac syndrome have been published. Encephalopathy is consistently reported as one of the most common findings at presentation [4, 9–13]. The nature of reported cognitive deficits at presentation arising as part of the encephalopathy is heterogenous and includes confusion [10, 14, 15], inattention [12], slowed processing speed, executive dysfunction [16], memory impairment [11–13, 17] and aphasia [13, 18]. Some patients display almost none of these symptoms of cognitive impairment whereas others are so profoundly affected as to be stuporous.

When attempting to quantify the nature and extent of cognitive impairment in Susac syndrome, there is a significant challenge in comparing findings between case series due to a lack of standardised assessments of cognition at disease onset and throughout the disease course. The timing of cognitive assessments is also variable in relation to disease onset, remission and relapses, which limits the generalisability of findings. In addition, there is a reliance on imprecise clinical descriptors in the published literature in which broad, unquantified descriptive terms such as “cognitive impairment” are used, which lack sufficient rigor for meaningful interpretation.

A small number of case reports have detailed the cognitive profile at initial presentation with standardised assessments, including the Montreal Cognitive Assessment (MoCA) [19, 20], Mini-Mental State Examination [21] and the Addenbrooke’s Cognitive Examination [21, 22]. Based on these few data, it appears that cognitive impairment at disease onset is heterogenous in terms of severity and domains affected. However, it is not possible to reliably extrapolate patterns of disturbance from these small samples using relatively brief screening tools, which do not readily enable between-domain comparisons.

The longitudinal trajectory of cognitive impairment and recovery is also poorly understood. To date, the largest study reporting cognitive outcomes in Susac syndrome includes 11 participants with comprehensive neuropsychological testing completed two years apart [23]. Impairments were most commonly seen in the domains of attention, executive functioning, and language, which all improved at 24 months following treatment with various combinations of aggressive immunotherapy [23]. Another cohort study assessed 10 patients followed up for an average of 20 months; four patients had a MoCA score of less than 27 with executive function and short-term recall most affected, and orientation and naming relatively preserved [11]. Despite the presence of persistent deficits, the study did not indicate whether there was a trend towards improvement. A few case reports also detail more comprehensive neuropsychological longitudinal data and indicate an overall improvement in cognition over time, albeit with ongoing deficits in aspects of executive function at approximately two years follow-up [21, 22, 24]. Other studies have shown chronic impairments in attention [22, 25], visuospatial construction [22, 24, 25], encoding [24] and memory [25]. These preliminary findings highlight the potential inter-individual variability in the cognitive phenotype and the nature of cognitive recovery in Susac syndrome.

Despite these limited data, important questions concerning cognition in Susac syndrome remain unanswered, including the correlation between cognitive performance and disease activity, as well as cognitive function in response to treatment and the influence of brain lesion extent and location. Cerebral lesions in Susac syndrome can affect almost any area of the brain including the meninges, cortical grey matter, deep and periventricular white matter, corpus callosum, basal ganglia, thalamus, brainstem, and cerebellum [26]. The pattern and severity of cognitive impairment in Susac syndrome may reflect which, and how many, of these structures are affected in any individual patient, and may reflect global lesion load or the extent of involvement of critical brain regions such as the cortex and/or subcortical white matter tracts including the corpus callosum. Recent work using 3T MRI has shown that even normal appearing white matter is subject to diffuse microstructural injury in Susac syndrome [27].

Preliminary data support cognitive recovery in conjunction with immunotherapy, indicating an apparent treatment effect [21, 23]. The possibility of persistent cognitive impairment emphasises the need for early diagnosis and immunotherapy to mitigate the chance of long-term cognitive disability associated with Susac syndrome.

There is a limited understanding of how longitudinal cognitive outcomes are associated with underlying pathology as observed on neuroimaging. Global and regional (namely corpus callosal) brain atrophy in Susac syndrome appears to progress independently of relapse or treatment [16]. Unexpectedly in this study, cognitive performance—which included long-term deficits in processing speed and executive function – did not correlate with the severity of atrophy [16]. The mechanisms for the disjunction between anatomical and functional pathology are unknown, although sample size and absence of a healthy control group for comparison are acknowledged as study limitations.

Therefore, there is a need for standardised and more comprehensive cognitive assessment in larger cohorts to 1) clarify the nature of the cognitive impairment associated with Susac syndrome at disease onset and longitudinally, 2) understand the impact of treatment on cognitive functions with the view to improve long-term outcomes, and 3) correlate the clinical cognitive profile with structural and functional neuroimaging, to better understand the underlying pathophysiological mechanisms of Susac syndrome. Standardised cognitive assessments should be inclusive of key cognitive domains (attention, processing speed, memory, language, and visuospatial function) using validated psychometric tests [23]. Precedent exists for comparable screening tools established for use in multiple sclerosis; such examples include the Symbol Digit Modalities Test (SDMT) which measures information processing speed, lexical access speed and memory [28], and the Brief International Cognitive Assessment for Multiple Sclerosis (BICAMS), validated for use internationally to assess global cognitive function and more specifically information processing speed, and immediate verbal and visual recall [29]. It has been proposed that the BICAMS may serve as a model for use in other conditions as a global, collaborative initiative to improve cognitive screening in neurological disorders [29], and it is certainly conceivable that similar could be applied to Susac syndrome research.

### Psychiatric features associated with Susac syndrome

There is a paucity of data with respect to the psychiatric phenotypes associated with the disease onset and trajectory in Susac syndrome. The hallmark review published in 2013 implicated high rates of mental disorders, including emotional disturbance (16%), behaviour change (15%), apathy (12%), personality change (12%) and psychosis (10%) [7]. Subsequent case series have not collected data systematically in relation to psychiatric phenomenology or syndromes. Frequently, there is reference to non-specific emotional and behavioural disturbance, such as “behavioural change” [14, 30], “behavioural, conduct or mood disorder” [11], and simply “psychiatric” symptoms [13]. With respect to defining the behaviour change, reports in the last decade ranged from apathy [12, 16] to agitation [17], disinhibition and aggression [13]. Although this is sufficient to meet the clinical criteria for “brain involvement” according to the EuSaC diagnostic criteria [8], such terms do little to elucidate the nature of psychopathology associated with Susac syndrome. Moreover, as the terms do not clearly correspond with any psychiatric research or clinical diagnostic classification system, it is unclear if the behaviour change occurs as part of a recognisable psychiatric syndrome, such as major depressive disorder or bipolar disorder. This is important if we are to better understand the psychopathology associated with Susac syndrome, which in turn is required to better inform adjunctive (symptomatic) treatment.

Despite the absence of more detailed neuropsychiatric information from case series, insights into the psychiatric phenomena associated with Susac syndrome can be found in published case reports. Mood disorders have been associated with Susac syndrome, both at the time of presentation and developing during the disease course. One case report described the onset of mania associated with tapering of methylprednisolone and transition to rituximab; symptoms failed to respond to haloperidol and lorazepam but did respond to valproic acid as an adjunct to immunotherapy [31]. Another case report described the presentation of mania in the context of corticosteroid use for the treatment of Susac syndrome, which responded to cessation of corticosteroid treatment and the introduction of lithium and risperidone [32]. Whilst these presentations occurred in the context of active disease and/or treatment changes, there are two case reports, both of females in their late 30s, who developed bipolar disorder (with both manic and depressive episodes) 1–2 years after the initial presentation of Susac syndrome [33, 34]. Both had a significant family history of psychiatric illness reported, and both developed mood symptoms whilst the Susac syndrome itself was in remission. Based on the available literature, it is impossible to appreciate whether the relationship between bipolar disorder and Susac syndrome is causal, how it is influenced by corticosteroid treatment, or whether the conditions co-occurred by coincidence.

Cases of pseudobulbar affect have also been reported during the course of Susac syndrome, including involuntary crying at initial presentation [35], as well as uncontrollable laughing and crying following the commencement of methylprednisolone and intravenous immunoglobulin [19]. Although the pathophysiology of pseudobulbar affect is not well understood, its presentation in Susac syndrome could be explained by lesions involved in the complex neurocircuitry of emotional control, classically the cortico-ponto-cerebellar circuit, as was implicated in both case reports.

Positive symptoms of psychosis have also been implicated in the presentation of Susac syndrome, including auditory and visual hallucinations, persecutory delusions and thought disorder [36]. In the absence of a description of cognitive function, one cannot ascertain whether this presentation was consistent with primary psychosis, or secondary to delirium. Moreover, this case described a history of comorbid cannabis dependence and stimulant misuse, although it is reported that substance intoxication and withdrawal were “ruled out” as causal. Oral haloperidol was commenced in addition to prednisone and cyclophosphamide, with slow resolution of psychosis. Marked anxiety has also been implicated [37, 38], including in a young male with a history of heavy cannabis use and sporadic amphetamine use [37]. Another intriguing observation relates to cases of Susac syndrome occurring following use of cocaine adulterated with levamisole [39, 40]. Levamisole, an antihelminthic drug, now used only in veterinary medicine, has been reported as a trigger for a range of inflammatory and non-inflammatory vasculitides [40]. It remains to be determined whether substance use disorders are a risk factor for the development of Susac syndrome and how it may influence concurrent psychopathology.

Thus, from the limited literature available, it appears that the psychiatric manifestations are diffuse, and give rise to several pertinent questions. Firstly, it is unclear what proportion develop transient psychiatric symptoms as part of an encephalopathy, in contrast to what proportion develop a diagnosable psychiatric syndrome. Secondly, it is also unclear as to whether psychiatric manifestations are a direct result of the disease process, or a secondary phenomenon. Finally, it is unclear as to how pre-existing substance use and psychiatric comorbidity might influence the presentation of psychopathology in Susac syndrome, as well as the longitudinal course. Treatment guidelines for Susac-associated psychopathology are also lacking; whether specific symptomatic treatment with psychotropics, in addition to immunotherapy, is needed and if so, what duration of treatment is optimal, requires clarification.

## Conclusions and future directions

Susac syndrome is a rare but important differential diagnosis for a range of neurological and psychiatric presentations. It is commonly misdiagnosed, which is of critical relevance given the importance of prompt treatment on long-term outcomes. Although neuropsychiatric manifestations are common, they remain poorly characterised. There is an important need to address this knowledge gap. A proposed method is outlined in Table 1.

**Table 1:** Proposed clinical and research priorities for addressing the neuropsychiatric features of Susac syndrome

Domain	Goals	Proposed strategies
Phenotyping	• Clarify the nature of neuropsychiatric features in Susac syndrome • Identify those at risk of developing neuropsychiatric symptoms in Susac syndrome	• Development of a proposed standardised battery evaluating cognitive and psychiatric symptoms at presentation and longitudinally • Development of a multisite, international registry • Development of a minimum dataset for application by registry participants
Pathophysiology	• Determine if neuropsychiatric symptoms in Susac syndrome are directly related to the disease process or secondary to the functional impacts of the illness and treatments	• Identify reliable biomarkers (peripheral blood, neuroimaging, CSF, and histopathology), and investigate the relationship between clinical phenotypes and these biomarkers
Treatment	• Develop an evidence-based therapeutic guideline for Susac syndrome, which takes into consideration cognitive and psychiatric outcomes • Understand the role of psychotropic medication in targeting neuropsychiatric symptoms in Susac syndrome	• Development of a multicentre, international patient registry which involves capture of a minimum data set on treatment and neuropsychiatric outcomes • Investigate the correlation between the nature/dose/duration of treatment and cognitive and psychiatric outcomes
Long-term outcomes	• Better understand the cognitive and psychiatric prognosis of Susac syndrome • Understand the predictors of long-term cognitive and psychiatric outcomes in Susac syndrome	• Inclusion of cognitive, psychiatric, and functional assessments longitudinally

Future studies will require systematic data collection to better enable phenotyping of the cognitive and behavioural components of the condition. The data need to be captured at the time of presentation and at standardised longitudinal timepoints, and in response to treatment. Ideally, this should involve development of a standardised battery evaluating cognitive and psychiatric symptoms, which can be administered on presentation and then serially, with a minimum dataset identified. Given the low prevalence of the condition, future research endeavours would benefit from collaboration and a multicentre international registry, which includes a minimum data set of cognitive and psychiatric features for harmonisation across centres. This will also enable further important questions to be addressed, including whether cognitive and psychiatric symptoms arise as a direct result of the pathological process of the disease itself, or secondary to the psychological and functional impacts of the condition.

Blueprints for an international registry are well-established for other neuroimmunological conditions. “MSBase”, an international, online collaborative, was established in 2004 to improve outcomes in multiple sclerosis and other neuroimmunological diseases [41]. To date, the registry has amassed more than 100,000 participants from 45 countries globally [42]. Further to this, the Big Multiple Sclerosis Data (BMSD) Network has addressed technical, ethical and legal challenges to showcase the feasibility of harmonising international registries [43]. Clinicians and researchers could leverage existing infrastructure to establish an international Susac syndrome registry in parallel, using standardised data capture programs such as Research Electronic Data Capture (REDCap) which are widely accessible internationally.

To better understand the pathophysiology of neuropsychiatric symptoms in Susac syndrome, reliable biomarkers of the condition need to be identified and investigated in relation to psychopathology. In addition to the well-described “snowball” lesions of the corpus callosum on MRI [44] and elevated total protein in cerebrospinal fluid found in the majority (> 80%) of patients diagnosed Susac syndrome [45], preliminary findings suggest that elevated serum neurofilament light chain and serum glial fibrillary acidic protein could be useful biomarkers of disease activity and treatment efficacy [46, 47]. Future prospective studies should consider these biomarkers in relation to cognition and psychiatric symptoms, and look at whether they predict clinical and functional outcomes.

Questions also arise as to the optimum treatment approach: is immunotherapy itself sufficient to lead to the remission of neuropsychiatric symptoms, or should immunotherapy be augmented with psychotropics? If so, which psychotropics, and for what duration? Moreover, studies are required to ascertain the effectiveness of cognitive rehabilitation and of psychological therapies, such as cognitive behavioural therapy, to manage symptoms.

As a rare disease, the challenges in studying Susac syndrome are substantial but no less rewarding than studying more common diseases. This is another reason why a multisite registry would provide an invaluable opportunity to study long term outcomes, including the relationship between disease burden, neuropsychiatric symptoms, different treatments, and psychopathology, and to provide insights into functional recovery.

## Data Availability

Data sharing is not applicable to this article as no datasets were generated or analysed in this study.
